# The complete chloroplast genome sequence of *Knema conferta* (Myristicaceae)

**DOI:** 10.1080/23802359.2020.1835574

**Published:** 2020-11-13

**Authors:** Feng-Liang Zhang, Tian Yang, Chang-Li Mao, Xiao-Qin Li, Qi Zhao, Yu Wu

**Affiliations:** Yunnan Institute of Tropical Crops, Xishuangbanna, China

**Keywords:** *Knema conferta*, chloroplast genome, Myristicaceae

## Abstract

*Knema conferta* is a member of Myristicaceae. The *K. conferta* chloroplast genome is found to be 155,744 bp in length and has a base composition of A (30.02%), G (19.30%), C (19.90%), and T (30.78%). The genome contained two short inverted repeat (IRa and IRb) regions (48,052 bp) which were separated by a large single copy (LSC) region (86,926 bp) and a small single copy (SSC) region (20,770 bp). The genome encoded a total of 128 unigenes, including 89 protein-coding genes, 31 transfer RNA (tRNA) genes, and 8 ribosomal RNA (rRNA) genes. Further, complete chloroplast sequence of *K. conferta* was aligned together with 2 species of Knema and 5 basal angiosperms species which have reported the complete chloroplast sequence. This complete chloroplast genome will provide valuable information for the development of DNA markers for future species resource development and phylogenetic analysis of *K. conferta*.

*Knema conferta*, belongs to Knema of Myristicaceae, which is distributed from the Malay Peninsula to the Indochina Peninsula, and Cangyuan County, Yunnan Province, China (Editorial Committee of Chinese Academy of Sciences Flora 1979). So far, there are no reports about the research on this species. In this study, we characterized the complete chloroplast genome sequence of *K. conferta* for phylogenetic analysis. The annotated genome sequence has been deposited Genbank under the accession number MN683754.

The fresh leaves of *K. conferta* was collected in 2017 from Nangunhe River valley, Yunnan, China (99°01.013′E, 23°16.943′N). The tree from which we collected specimens was preserved in situ and the number of voucher specimen is 20161101. The genome DNA of *K. conferta* was extracted using the DNeasy Plant Mini Kit (QIAGEN, Valencia, CA), and its remaining DNA was stored in an ultra-low temperature freezer now. Genome sequencing was performed using Roche/454, sequencing libraries were prepared by the GS Titanium library preparation kit. The chloroplast genome assembled using CLC Genomic Workbench v3.6 (http://www.clcbio.com). The genes in the chloroplast genome were predicted using the DOGMA program (Wyman et al. 2004).

The circular genome is 155,744 bp in size, and comprises a large single copy (LSC) region (86,926 bp), a small single copy (SSC) region (20,770 bp), and two short inverted repeat (IRa and IRb) regions (48,052 bp). The base composition of the circular chloroplast genome is A (30.02%), G (19.30%), C (19.90%), and T (30.78%). The GC content of whole *K. conferta* chloroplast genome was 39.20%. The chloroplast genome has 89 protein-coding genes, 31 transfer RNA (tRNA) genes, and 8 ribosomal RNA (rRNA) genes.

To study *K. conferta* phylogenetic relationship with the angiosperms, *Knema furfuracea* (Tian et al. 2020) and *Knema elegans* of Myristicaceae (ChangLi et al. 2020) and other complete chloroplast genome sequences of angiosperms were download for analyses. The maximum likelihood phylogenetic was performed using MEGA X (Kumar et al. 2018) (Figure 1). A bootstrap analysis was performed on the resulting phylogenetic tree, and values were obtained after 1000 replications. The result shows that *K. conferta* was clustered with other species and closely to *Knema furfuracea* and *Knema elegans* chloroplast complete genome.

**Figure 1.**
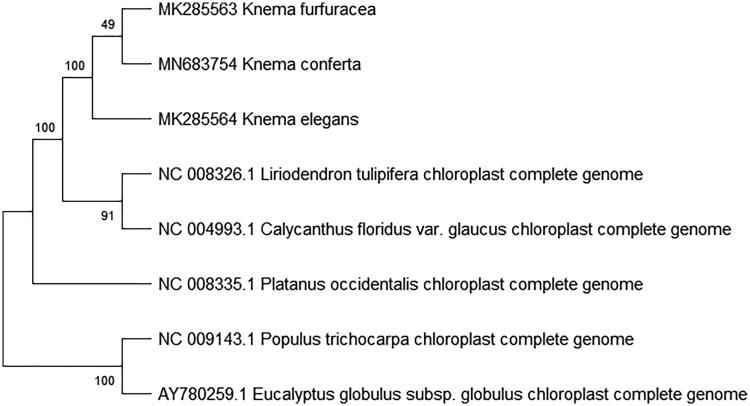
Maximum likelihood phylogenetic tree of K. conferta with 7 species based on complete chloroplast genome sequences. The gene’s accession number is list in figure and the data of K. elegans and K. furfuracea come from author.

The complete chloroplast genome of *K. conferta* would provide information on development of molecular markers and phylogenetic analysis in the future.

## Data Availability

The data that support the findings of this study are openly available in GenBank at https://www.ncbi.nlm.nih.gov/, reference number MN683754.
